# Cytoskeletal Dependence of Insulin Granule Movement Dynamics in INS-1 Beta-Cells in Response to Glucose

**DOI:** 10.1371/journal.pone.0109082

**Published:** 2014-10-13

**Authors:** Aoife T. Heaslip, Shane R. Nelson, Andrew T. Lombardo, Samantha Beck Previs, Jessica Armstrong, David M. Warshaw

**Affiliations:** University of Vermont, Department of Molecular Physiology and Biophysics, Health Sciences Research Facility, Burlington, Vermont, United States of America; J. Heyrovsky Institute of Physical Chemistry, Czech Republic

## Abstract

For pancreatic β-cells to secrete insulin in response to elevated blood glucose, insulin granules retained within the subplasmalemmal space must be transported to sites of secretion on the plasma membrane. Using a combination of super-resolution STORM imaging and live cell TIRF microscopy we investigate how the organization and dynamics of the actin and microtubule cytoskeletons in INS-1 β-cells contribute to this process. GFP-labeled insulin granules display 3 different modes of motion (stationary, diffusive-like, and directed). Diffusive-like motion dominates in basal, low glucose conditions. Upon glucose stimulation no gross rearrangement of the actin cytoskeleton is observed but there are increases in the 1) rate of microtubule polymerization; 2) rate of diffusive-like motion; and 3) proportion of granules undergoing microtubule-based directed motion. By pharmacologically perturbing the actin and microtubule cytoskeletons, we determine that microtubule-dependent granule transport occurs within the subplasmalemmal space and that the actin cytoskeleton limits this transport in basal conditions, when insulin secretion needs to be inhibited.

## Introduction

Insulin secretion from pancreatic β-cells is critical for proper maintenance of blood glucose levels, with perturbations to this process leading to diabetes [1]–[3]. Upon glucose stimulation, insulin release from dense core granules occurs in two phases, the first, rapid phase involves a “readily releasable pool” of stably docked granules at the plasma membrane whereas the second, prolonged phase occurs in response to sustained stimulation and involves mobilization of a “reserve pool” of granules in close proximity (i.e. within 200 nm) of the plasma membrane [4]. In general, secretary granule delivery to the plasma membrane relies on long distance transport by kinesin-1 molecular motors on microtubules from the granule synthesis site in the trans-golgi network to the cell periphery [5]. Once at the periphery, granules are believed to be handed off to myosin Va motors for distribution and retention within the actin-rich cell cortex adjacent to the plasma membrane [6]–[8]. Evidence for molecular motor function being critical for granule transport is based on perturbation of either kinesin-1 or myosin Va function by expression of dominant negative constructs or knockdown of protein levels with siRNA resulting in diminished insulin release, loss of directed granule movement, and altered granule localization at the plasma membrane [7], [9]–[14]. Implicit in this model is that the microtubule and actin networks serve as cytoskeletal tracks upon which the motors transport their granule cargos. However, the actin and microtubule cytoskeletons may not be passive players but actively involved in modulating insulin granule trafficking.

Two differing models exist for how the filamentous state of the β-cell actin cytoskeleton influences insulin secretion. In the first model, the F-actin cortex may act as a physical barrier to secretion by trapping granules in the dense actin meshwork and preventing granule movement to the plasma membrane [15]–[18]. Consistent with this “barrier” model was the observed increase in insulin secretion following F-actin depolymerization by treating cultured cells and pancreatic islets with latrunculin and cytochalasins [18]–[20]. Therefore, cortical actin remodeling would be necessary to eliminate the effective barrier to granule movement upon glucose stimulation [21]. As further support, preventing potential actin remodeling by stabilizing the actin cytoskeleton with jasplakinolide, greatly inhibits insulin secretion [22]–[24]. However, jasplakinolide treatment has been reported to enhance secretion as well [17], [20], [25]. In contrast to the “barrier” model, a recent model suggests that under basal conditions and during the first phase of insulin secretion following glucose stimulation, cortical actin is maintained in the globular, G-form by the F-actin severing protein, cofilin [26]. During the second, prolonged phase of secretion, activity of the N-WASP-Arp2/3 complex results in actin polymerization and branched F-actin formation to facilitate trafficking of granules to the plasma membrane. Thus, the filamentous state of the actin cortex and its modulation of insulin granule trafficking remain controversial.

The microtubule network acts as tracks for granule delivery by kinesin-1 to the cell periphery and microtubule depolymerization by various drugs (e.g. colchicine, nocodazole) reduced granule movement and insulin secretion in whole tissue or tissue culture systems [14], [27]–[29], whereas taxol stabilization of microtubules resulted in no effect [27] or inhibition of insulin secretion [30], [31]. However, a recent report of GLUT-4 vesicle trafficking in adipocytes suggests that microtubule organization and dynamics in response to insulin stimulation may be involved in spatially dictating plasma membrane sites of vesicle fusion [32]. Thus, microtubules may not be simply passive tracks for granule delivery.

To determine if the actin and microtubule cytoskeletons have multiple roles in granule trafficking, we used total internal reflectance fluorescence (TIRF) microscopy and super-resolution imaging by stochastic optical reconstruction microscopy (STORM; reviewed in [33]) to visualize individual actin filaments and microtubules in the cortex of cultured pancreatic β-cells (INS-1) [34]. In addition, the movement dynamics of individual insulin granules within 150 nm of the plasma membrane (i.e. within the TIRF field) was observed with high spatial and temporal resolution before and after glucose stimulation and after pharmacologic perturbations of the cells’ actin and microtubule cytoskeletons. In basal, low glucose conditions, when insulin granule secretion must be limited, directed microtubule-based motion is limited by the actin cytoskeleton in the subplasmalemmal space. In stimulatory conditions where the granules must be mobilized to the plasma membrane, microtubule-based directed motion increases without rearrangement of the actin cytoskeleton. Collectively, our data suggest that in addition to the established role of microtubules in long distance transport from the cell center, microtubules are needed for directed granule transport near the plasma membrane. It appears that the actin cytoskeleton in the subplasmalemmal space regulates this directed transport, presumably to prevent granules from reaching the plasma membrane for insulin secretion in basal, low-glucose conditions.

## Materials and Methods

### INS-1 cell culture

INS-1 cells [34] between passage 50 and 65 were maintained in culture at 37°C, 5% CO_2_ in RPMI culture media (RPMI supplemented with 11.1 mM glucose, 10 mM Hepes, 10 mM glutamax, 1x antibiotic-antimyotic, 10 mM pyruvate, 50 µM tissue culture grade beta-mercaptoethanol (all purchased from LifeTechnologies, Grand Island NY). Before live cell imaging or STORM sample preparation, cells were plated onto poly-d-lysine coated 35 mM, #1.5 coverslip glass bottom culture dishes (MatTek Corporation, Ashland MA). After 24 hours normal RPMI culture media was replaced with RPMI media containing 2.8 mM glucose and grown for a further 24 hours. Cells were equilibrated in low glucose hanks buffered salt solution (HBSS: 114 mM NaCl, 4.7 mM KCl, 1.2 mM KH_2_PO_4_, 1.16 mM MgSO_4_, 20 mM Hepes, 2.5 mM CaCl_2_, 25 mM NaHCO_3_, 2.8 mM glucose, 2% BSA, pH 7.2) for 2 hours at 37°C. To stimulate with glucose HBSS containing 90 mM glucose was added to dishes so final glucose concentration was 20 mM before either fixation for immunofluorescence or transfer to the microscope for live cell imaging (see below). For drug treatments cells were equilibrated in low glucose HBSS for 90 minutes before drug addition and then incubated a further 30 minute in HBSS plus drug. Cells were stimulated as above in high glucose HBSS plus drug. Unless otherwise noted final drug concentrations were as follows: 2 µM cytochalasin D (Sigma-Aldrich, St. Louis, MO), 1 µM jasplakinolide (EMD Millipore, Billerica, MA), 3 µM, nocodazole (Sigma-Aldrich, St. Louis, MO), 2 µM, taxol (Cytoskeleton Inc., Denver CO).

### INS-1 cell transfection

INS-1 cells were transfected using lipofectamine 2000 reagent (LifeTechnologies, Grand Island, NY) as per manufacturer’s instructions using RPMI culture media to dilute reagents. 3.75 µl of lipofectamine was used per 1 µg of plasmid. The proinsulin-eGFP plasmid [35] was a kind gift from David Piston, Vanderbilt University. EB3-eGFP plasmid was previously published in [36] and GFP-tubulin plasmid was obtained from Clontech.

### Insulin Granule Imaging, Tracking, and Movement Analysis

#### Granule Imaging and Tracking

Cells were imaged in pre-warmed HBSS media on a Nikon Ti-E inverted microscope with a 100×1.49 NA objective in an environmental chamber heated to 37°C equipped with an Andor iXon X3 EMCCD camera (Andor Technology, Belfast, UK). Proinsulin-eGFP, GFP-tubulin and EB3-GFP samples were imaged using total internal reflectance microscopy excited with a 488 nm solid state laser (Andor Technology, Belfast UK) and emission collected at 525 nm at 19 fps, 10 fps and 5 fps, respectively. Images were collected in basal, low glucose conditions (2.8 mM glucose) before stimulation with glucose to a final concentration of 20 mM. Cells were imaged between 3 and 20 minutes after glucose addition. Insulin granules’ motions were tracked using the Image J v1.43 plug-in, SpotTracker2D (National Institutes of Health), allowing us to track the granules position at sub-pixel resolution in every frame of the movie so that mean square displacement (MSD) analysis could be performed (see below). Out-of-focus granules and granules which could not be spatially resolved from one another were eliminated from analysis. EB3-GFP point-to-point velocities were determined using the ImageJ 1.43 plug-in, MTrackJ.

Imaging and tracking of insulin granules in fixed cells were used as a control for stage drift in our imaging set-up and to determine our imaging resolution. To prepare these fixed cells, INS-1 cells were transfected with proinsulin-eGFP as described above. 24 hours after transfection cells were fixed with 4% paraformaldehyde in 1×PBS for 15 minutes at room temperature. Cells were washed three times in 1×PBS and stored at 4°C. Samples were first imaged by maximum projection through time to visually assess for the occurrence of stage drift. MSD analysis was subsequently performed and alpha values (diffusive exponent, see below) for fixed samples was determined to be 0.05, indicating negligible stage drift. To determine our tracking spatial resolution, granules were imaged and tracked as with live cells. Tracking resolution of 23 nm was defined as the standard deviation of the X and Y coordinates from stationary granules in fixed cells.

#### Determining vesicle number at the cell cortex

The number of vesicles in the TIRF field of each cell was counted using the count tool in Adobe Photoshop. The imaged cell area was then determined using the measure tool in ImageJ v1.43. Reported vesicle numbers were normalized to the imaged cell area in µm^2^. To determine if vesicle number changed upon glucose stimulation in control and drug treated cells a two way ANOVA was performed using GraphPad Prism v6.01.

#### Mean Squared Displacement (MSD) Analysis

MSD analysis [37] was calculated according to:
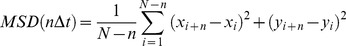
(1)Where *N* is the total number of frames in the trajectory, *n* is the number of frames for different time intervals, *Δt* is the time interval between frames for the trajectory (typically 52 ms), and (*x_i_,y_i_*) is the position of the insulin granule at time *i*. Individual trajectories are classified as “Stationary,” “Diffusive-like,” or “Directed” based on their “Diffusive Exponent” or α value, according to the generalized form, *MSD*∼*nΔt^α^*, which is determined via linear regression to plots of log(*MSD*) vs log(*nΔt*). Within diffusive periods, diffusion coefficients were calculated from the slope the MSD plot, according to:



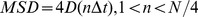
(2)As some trajectories contained visually apparent changes in the types of motion (e.g. a long directed run followed by a stationary period), we developed and applied a “ChangePoint” algorithm attributed to [38], which seeks to identify transitions between distinct modes of motion within a trajectory. Trajectories that contained a change in the type of motion were segmented into their component modes utilizing multiple iterations of the ChangePoint algorithm. This ChangePoint approach seeks to identify transitions in the type of motion through optimization a goodness-of-fit measure (Baysian Information Criterion, BIC) of linear fits to log-scaled MSD plots for the trajectory segments before and after a candidate transition. For a detailed explanation and validation of the ChangePoint algorithm, see Methods S1 and Figures S3 and S4 in File S1. MSD and ChangePoint analysis were performed with custom scripts written in R, a programming language and environment for statistical analysis.

### Deconvolution epifluorescence microscopy

To image microtubules throughout the cell volume 3D image stacks were collected at z-increments of 0.5 µm using an Applied Precision Delta Vision imaging station constructed on an Olympus inverted microscope base with a 100× oil immersion lens (1.4 NA). Deconvolved images were computed using the point-spread functions and software supplied by the manufacturer.

### Super-resolution imaging sample preparation

To image actin filaments and microtubules in INS-1 cells, cells were grown on MatTek dishes and stimulated with glucose as described above. For actin labeling cells were fixed and permeabilized as previously described [39]. To stain actin filaments, samples were incubated with 0.5 µM AlexaFluor647-phalloidin (LifeTechnologies, Grand Island, NY) overnight at 4°C. Immediately before imaging, cells were washed x 2 in 1×PBS and incubated in 2 ml of STORM imaging buffer (50 mM Tris-HCl pH 8.0, 10 mM NaCl, 10% glucose, 5 mg/ml glucose oxidase, 0.35 mg/ml catalase and 10 mM cysteamine (MEA) (Sigma, St. Louis MO). To image microtubules samples were fixed and stained as previously described [40]. Monoclonal mouse anti-tubulin antibody (Sigma, St. Louis MO) was diluted 1∶500 in 1×PBS, 2% BSA and secondary antibody goat anti-mouse AlexaFluor647 (A21236, LifeTechnologies, Grand Island, NY) was diluted 1∶500 in 1×PBS, 2% BSA. Samples were post-fixed in 3% formaldehyde, 0.1% glutaraldehyde in 1×PBS for 10 minutes before being washed x3 in 1×PBS. Samples were stored in 1×PBS at 4°C.

### STORM Image data acquisition and analysis

Super-resolution images were acquired using Nikon’s N-STORM super-resolution microscope system. The 647-nm laser was used to excite fluorescence from Alexa Fluor 647 (∼15 kW/cm^2^), with the 405-nm laser used to reactivate the fluorophores from the dark state back to the emitting state. Images were acquired at 66 Hz. 75,000 frames were recorded to generate the final super-resolution microtubule images and 125,000 frames for the actin images. Images were analyzed using Nikon’s imaging software NIS-elements with N-STORM (Nikon Instruments, Melville, NY). Fluorophores with an axial ratio greater than 1.3 were eliminated from analysis. Minimum and maximum intensity thresholds were determined on a movie by movie basis but spatially resolved fluorophores with peak intensity of less than 800 photons were typically eliminated from analysis. All images were drift corrected using NIS elements software. To quantify the number of actin intersections and the density of the actin cytoskeleton, four 1 µm^2^ sections/cell were skeletonized using the line tool in Adobe PhotoShop CS5.1, then using either the counting tool in PhotoShop or the “measure” tool in Image J v1.43, the number of actin intersections and the actin density (i.e. total length of actin/µm^2^ of imaged cell area) was determined. To quantify the density of microtubule cytoskeleton, four 4 µm^2^ sections/cell were analyzed by creating a tiff-stack of the same image in ImageJ v1.43 and then using the Plug-in, MTrackJ, to keep track of microtubules for which end to end lengths have been measured in consecutive images. By this approach, the total length of microtubule/µm^2^ of cell image was obtained. Analysis was carried out on at least 6 independent cells.

### Statistics

Statistical significance for granule velocities was established using a t-test. Values are reported as mean ± Standard Error. For granule diffusion coefficients significance was determined using both t-test and KS-test on log-transformed data. Log-normally distributed diffusion coefficients are reported as geometric mean ± error factor. The error factor is defined as the square root of the width of the 90% confidence interval [41]. Statistical significance for EB3 velocities and actin and microtubule densities was determined using students t-test in GraphPad Prism.

## Results

### Insulin Granule Motion and Transport Dynamics under Basal and Glucose-Stimulated Conditions

**Figure 1:**
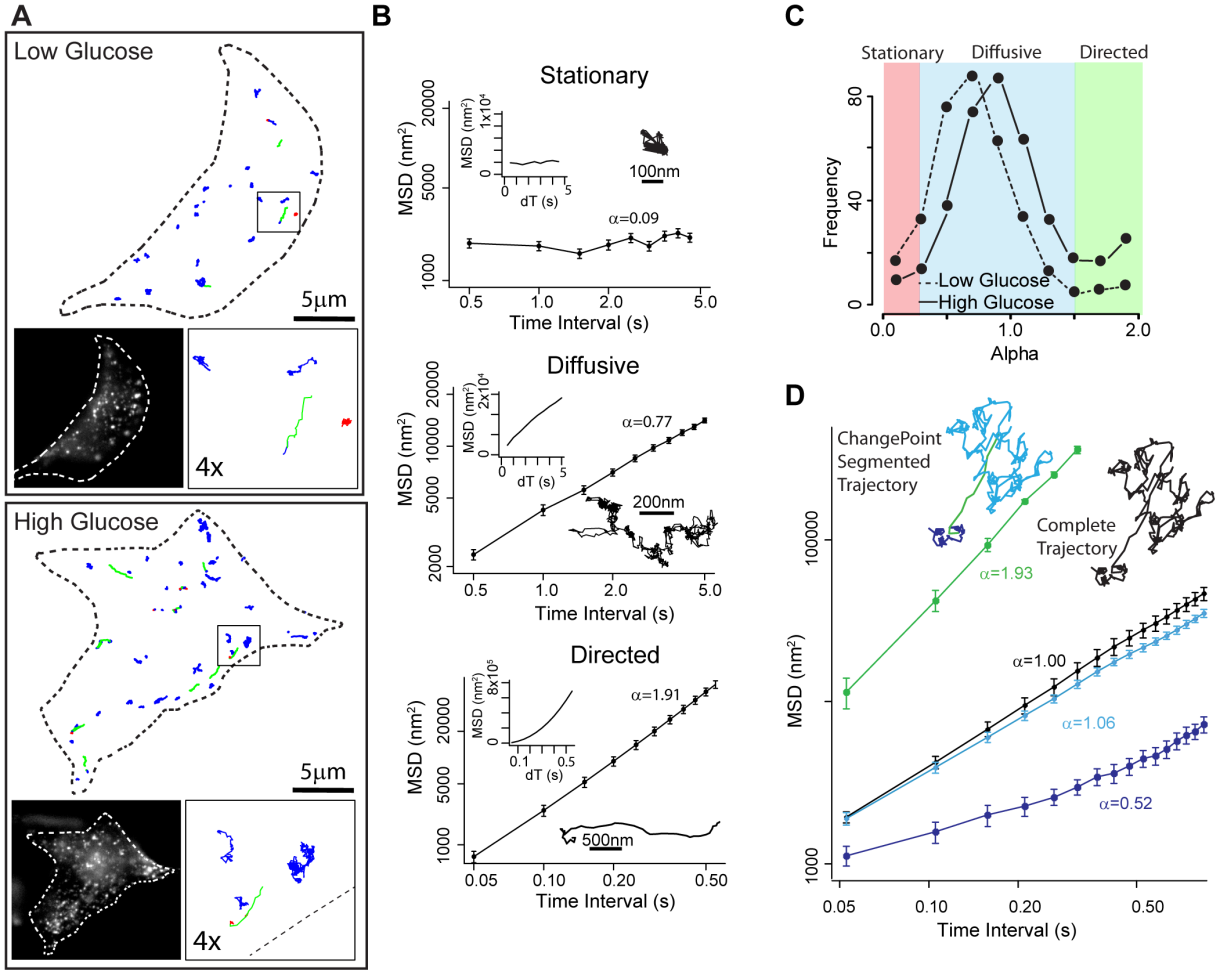
Modes of motion of GFP-labeled insulin granules. (A) Outline of representative INS-1 cells with insulin granule trajectories overlaid in low (upper panel) and high (lower panel) glucose conditions. Left inset: image of INS-1 cell with eGFP-labeled insulin granules. Right inset: 4× magnification of trajectories in the black box. Trajectories are color-coded to depict mode of granule motion. Red indicates stationary granule, blue indicates diffusive-like motion, and green indicates directed motion. (B) Insulin granules display 3 distinct modes of motion: stationary, diffusive-like and directed. Example trajectories for each mode of motion and MSD plots on log-log axes (large graphs) and linear axes (inset; dT equals time interval) are shown. Diffusive exponent, α, which is the slope of the log-log MSD indicated. (C) Frequency distribution of α values in low (dashed line: 383 granules in 14 cells) and high (solid line: 457 granules in 15 cells) glucose conditions. α<0.25 characterizes granules as stationary (red shaded area), 0.25≤α≤1.5 as undergoing random motion (diffusive-like, blue shaded area) and α>1.5 as undergoing directed motion (green shaded area). (D) Log-log MSD plots and trajectory of a granule that switches modes of motion during a trajectory. When MSD analysis is performed on the entire trajectory, α = 1 (black line, black trajectory). After analysis using the ChangePoint algorithm, 3 periods of motion are identified: diffusive-like (α = 0.53, dark blue line), directed (α = 1.93, green line) and diffusive-like (α = 1.06, light blue line). Error bars indicate standard error.

To determine the movement dynamics of insulin granules in response to glucose stimulation and following structural perturbations to the microtubule and actin cytoskeletons, we fluorescently labeled insulin granules in INS-1 cells by expressing a proinsulin-eGFP construct [35] and tracked the motion of granules within ∼150 nm of the cell surface membrane using TIRF microscopy (Movie S1, Figure 1A).

**Figure 2:**
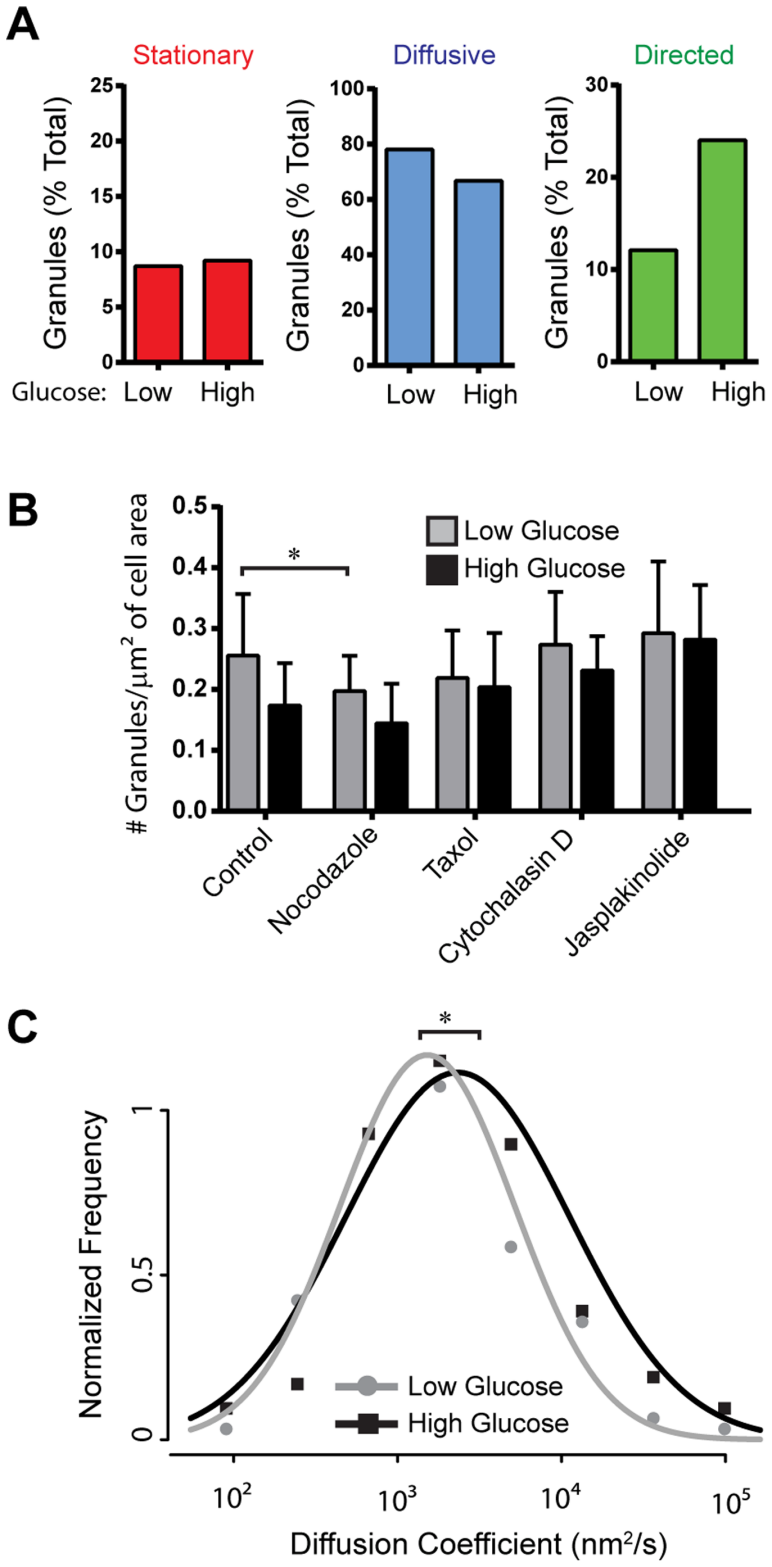
Effect of glucose stimulation on insulin granule motion. (A) Bar charts depict the percentage of granules that exhibit stationary, diffusive-like, and directed motions in low and high glucose conditions. 14 cells/383 granules in low glucose and 15 cells/457 granules in high glucose conditions were analyzed. (B) Effect of glucose stimulation on granule number at the subplasmalemmal space within the TIRF field. Glucose stimulation causes a significant decrease in granule number at the cell surface in control and drug treated cells as determined using a two-way ANOVA F(1, 148) = 8.81, p = 0.0035. In low glucose conditions nocodazole treatment results in a significant decrease in number of granules at the cell surface (p<0.05). Error bars indicate standard deviation. Number of cells analyzed in low and high glucose conditions, respectively, are as follows: control 15/16; nocodazole 21/24; taxol 19/20; cytochalasin D 13/15; jasplakinolide 6/7. (C) Normalized diffusion coefficient frequency distributions show that high glucose stimulation significantly increases of granule diffusion coefficients compared to basal, low glucose conditions (p<0.01).

Under basal conditions, 90% of tracked granules remained within the TIRF field during our standard 60 second period of movie acquisition (Movie S1). Granule movements were varied as depicted in x, y-displacement trajectories (Figure 1A and 1B). Most granules had diffusive-like motion while a small percentage were either stationary or underwent directed motion (Figure 1A and 1C, Movie S1). To characterize these modes of motion, trajectories were analyzed by mean square displacement (MSD) plots (See Materials and Methods; Figure 1B). On a log-log axis, the MSD slope defines the diffusive exponent, α, where α∼0 characterizes the granule as stationary, α∼1 as undergoing random motion (i.e. pure diffusion or diffusive-like motion due to either a granule being transported by motors stepping on a random cytoskeletal track [37] or being attached to a dynamic cytoskeletal element), and α∼2 as directed (e.g. motor-based transport) (Saxton, 1997). The MSD of individual granule trajectories resulted in a broad range of α between 0.25 and 1.5 with a peak near 0.75 (Figure 1C). To define our ability to distinguish between modes of motion by differences in α, we performed a control experiment to determine the inherent noise in all aspects of the imaging and motion data analysis. We fixed cells and tracked what by definition would be stationary granules (Figure S1 in File S1), which resulted in α = 0.05±0.02 (n = 40 trajectories from 6 cells). Using these raw data to define the imaging noise, we then generated model granule motion data that had defined stationary (α = 0), diffusive-like (α = 1), and directed (α = 2) motion with similar imaging noise added (Methods S1 and Figure S2 in File S1). Interestingly, MSD analysis of such model data gave α = 0.7±0.2 for purely diffusive-like motion and α = 1.9±0.1 for directed motion (Figure S2 in File S1). Based on the fixed cell experiment and these model data, we defined granules being stationary with α<0.25, diffusive-like motion with 0.25≤α≤1.5, and having directed motion with α>1.5 (Figures 1C, Figure S2 in File S1), similar to criteria used recently to parse the modes of early endosomal motion [42]. In addition, there was a number of granules which appeared to switch between modes of motion within a single trajectory (Figure 1D). MSD analysis of these “mode-switching” granules yielded alpha values which were not an accurate reflection of the modes of motion being exhibited by the granule (Figure 1D, black line). Therefore, to identify and characterize granules that exhibited more than one mode of motion, an unbiased ChangePoint algorithm was developed and used to analyze all granules (Material and Methods; Figure 1D and Figure S3 and S4 in File S1). Once granule motions were classified (i.e. stationary, diffusive-like, directed), under basal conditions the majority of granules displayed diffusive-like motion with a diffusion coefficient of 1514±94 nm^2^/s (Figures 1C, 2A and 2C; Table 1), similar to diffusion coefficients measured previously [22]. Only 12% of granules exhibited directed runs with a velocity of 1.19±0.12 µm/s while 9% were stationary (Figure 2A, Table 1). Following glucose stimulation, 45% fewer granules were present in the subplasmalemmal space (Figure 2B) but their motion was more dynamic (Figure 1A and 2A; Movie S1). 67% of the granules were still diffusive-like with a diffusion coefficient of 2325±129 nm^2^/s, significantly faster than in the basal state (Figure 2C and Table 1), while the percentage of granules undergoing directed runs doubled from 12% to 24% (Figure 2A).

**Table 1:** Granule diffusion coefficients (D) and velocities.

	Diffusion Coefficient (Mean±error nm^2^/s)		Velocity (Mean±SEM µm/s)	
	Low Glucose	High Glucose	Low Glucose	High Glucose
**Control**	1514±94	2325±129 *	1.19±0.12	1.16±0.08
**Nocodazole**	924±73 #	2393±106 *	N/A	N/A
**Taxol**	663±57 #	1616±86 * #	0.68±0.11 #	0.79±0.10 #
**Jasplakinolide**	998±68 #	1587±99 * #	N/A	1.10±0.17
**Cytochalasin D**	1491±97	3093±115 * $	0.71±0.09 #	0.98±0.10

Granule diffusion coefficients for diffusive-like motion and directed granule motion velocities for low and high glucose under various experimental conditions. “*” indicates that in diffusive populations, granules have significantly higher D upon glucose stimulation compared with low glucose samples (p<0.001, t test). “#” indicates diffusion coefficient or velocity are significantly lower than in control cells for the same stimulatory state (p<0.05, t test). “$” indicates that diffusion coefficient is significantly higher than in control cells for the same stimulatory state. Error term for D is defined as the square root of the 90% confidence interval [41].

### Cytoskeletal-Dependence of Granule Motion

To relate the observed modes of insulin granule movement to both the structure and dynamics of the cell’s cytoskeleton, we treated cells with either depolymerizing or stabilizing compounds that targeted either microtubules or actin filaments.

#### Microtubule Cytoskeleton

**Figure 3:**
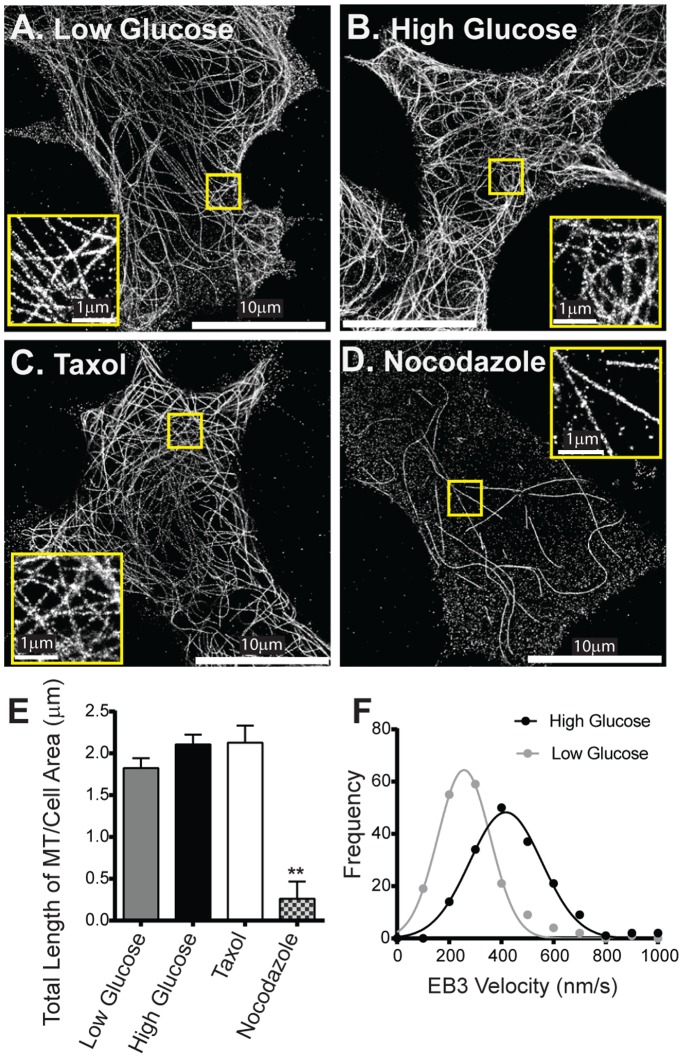
Effect of glucose stimulation on microtubule structure and dynamics. Super-resolution STORM images of microtubules in INS-1 cells in low (A) and high (B) glucose conditions, and after perturbation of the microtubule cytoskeleton with taxol (C) and nocodazole (D). Insets in A–D show 3× magnification of area in the yellow box. (E) Total microtubule (MT) length per µm^2^ of imaged cell area under various conditions. ** indicates p<0.001. (F) Frequency distribution of EB3 velocities show that glucose stimulation (black line: n = 170 from 6 cells) causes a significant (p<0.001) increase in microtubule polymerization velocities compared to low glucose conditions (grey line: n = 173 from 6 cells).

Under basal conditions, super-resolution STORM images of fixed INS-1 cells stained with anti-tubulin antibodies, show an extremely dense microtubule network with individual microtubules clearly resolved (Figure 3A). It is worth noting that these STORM images are obtained in TIRF, so that the visualized microtubules are within ∼150 nm of the cell surface membrane. However, this network exists throughout the volume of the cell as determined by deconvolution epifluorescence microscopy (Movie S2). The microtubule network was highly dynamic as visualized in live cells expressing GFP-tubulin (Movie S3), with microtubules undergoing cycles of growth and spontaneous shortening (i.e. catastrophe). Constant microtubule polymerization was confirmed by transfecting cells with EB3-GFP, a microtubule plus-end binding protein, which only binds to growing microtubule tips [43] (Movie S4). In response to glucose stimulation, the overall appearance of the microtubule network in STORM images was unchanged (Figure 3B) given that the total length of microtubule per µm^2^ of cell image was no different compared to basal conditions (1.8±0.06 µm vs 2.0±0.05 µm for low and high glucose, respectively) (Figure 3E). However, the dynamic nature of the microtubule network was enhanced as confirmed by an increased rate of microtubule polymerization estimated by faster EB3-GFP labeled microtubule tip velocities increasing from 256±3.7 nm/s to 416±4.8 nm/s upon glucose stimulation (p<0.001, Figure 3F; Movie S4).

**Figure 4:**
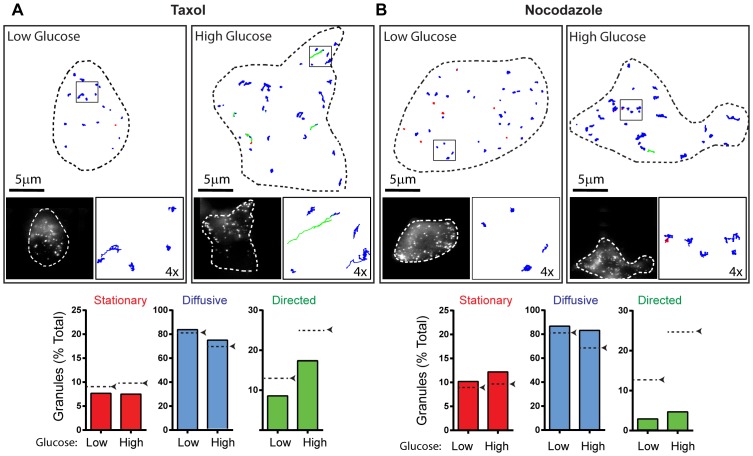
Effect of microtubule perturbation on insulin granule dynamics. (A and B: Upper Image Panels) Outline of a representative INS-1 cells with insulin granule trajectories overlaid in low and high glucose conditions after treatment with taxol (A) and nocodazole (B). Left inset: image of INS-1 cells with eGFP-labeled insulin granules. Right inset: 4× magnification of trajectories in the black box. Trajectories are color-coded to depict mode of granule motion. Red indicates stationary granule, blue indicates diffusive-like motion, and green indicates directed motion. (A and B: Lower Panel) Bar charts depict the percentage of granules exhibiting stationary, diffusive-like, and directed motions in low and high glucose conditions. Black arrows/dashed lines indicate the percentage of granules in each population in control cells from Figure 2A. Taxol: 17 cells/282 granules in low glucose and 15 cells/289 granules in high glucose conditions were analyzed. Nocodazole: 18 cells/321 granules in low glucose and 23 cells/428 granules in high glucose conditions were analyzed.

To determine if a dynamic microtubule network is critical to insulin granule trafficking, we stabilized microtubules by taxol treatment [44], as indicated by a diffuse EB3-GFP localization in cells due to the lack of growing microtubules (Movie S4). After taxol treatment, STORM images indicate that the total length of microtubule per µm^2^ of cell image remained unchanged at 2.1±0.14 µm (Figure 3C, 3E), which resulted in subtle effects on the distribution of granule modes of motion (Figure 4A, Movie S5). Most notably was the 30% decrease in the percentage of directed runs in both basal and stimulated conditions as well as their respective 1.5-fold slower transport velocities compared to untreated cells (Figure 4A; Table 1). In contrast, nocodazole treatment resulted in a significant loss of microtubules, as confirmed by STORM imaging with only 10% the total length of microtubules per µm^2^ of cell image remaining (Figures 3D and 3E). Nocodazole caused a modest, 20% decrease in the number of granules in the subplasmalemmal space in basal conditions, most likely due to an inhibition in the transport of new synthesized granules from the cell center during the 30 minute treatment time (Figure 2B). Of the granules remaining at the cell surface, microtubule depolymerization led to 4- and 6-fold reductions in the percentage of directed runs under basal and glucose stimulated conditions, respectively, compared to untreated cells (Figure 4B, Movie S6).

#### Actin Cytoskeleton

**Figure 5:**
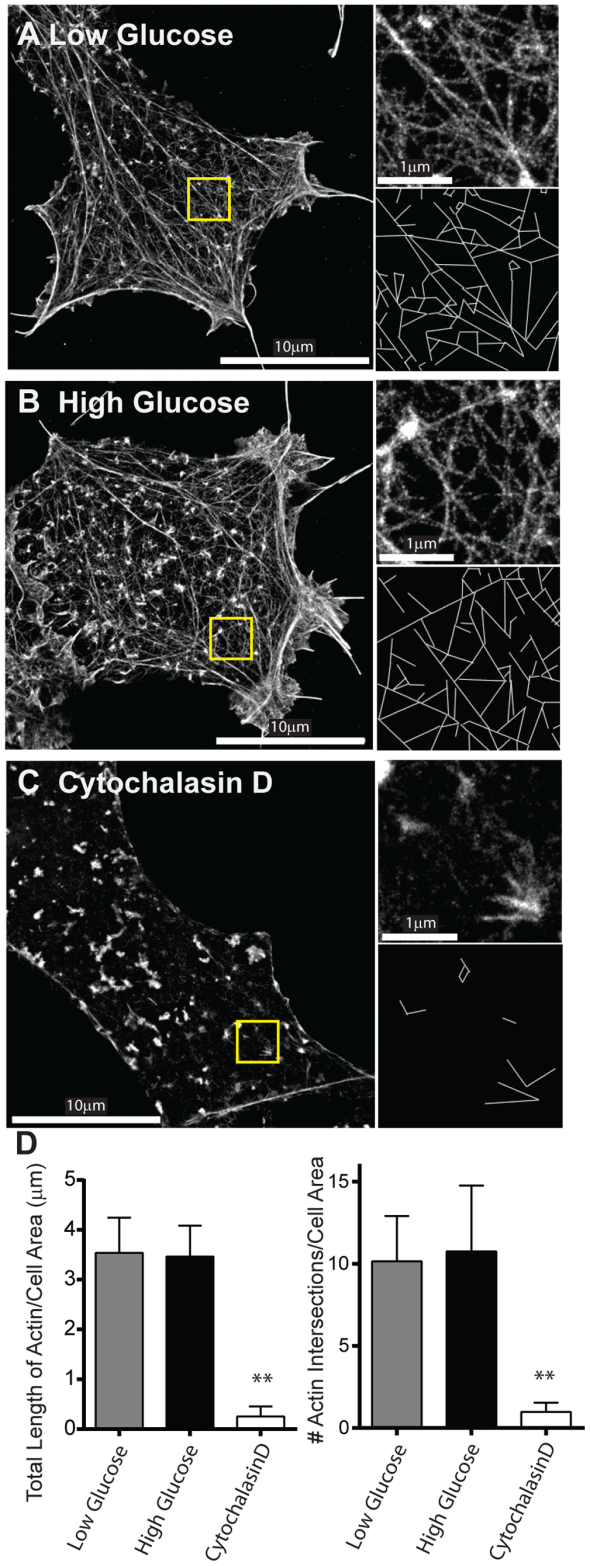
Effect of glucose stimulation on actin filament organization. Super-resolution STORM images of the actin cytoskeleton in INS-1 cells in low (A) and high (B) glucose conditions and after actin depolymerization with cytochalasin D (C). In each STORM image (A–C) yellow highlighted area is magnified 3x in the upper right image with skeletonized image below used for quantification of actin filament length and number of actin filament intersections in D. (D) Quantification of actin organization. Left: The length of actin per µm^2^ of imaged cell area under various conditions. Right: The number of actin intersections per µm^2^ of imaged cell area under various conditions. ** indicates p<0.001. Values are mean±SEM.

To image individual actin filaments within the subplasmalemmal space, STORM images of fixed INS-1 cells stained with AlexaFluor647-phalloidin were obtained [39]. In the basal state, a dense actin filament meshwork and apparent stress fiber-like actin cables were observed (Figure 5A). Since models suggest that the actin cytoskeleton acts as a barrier to granule delivery to the plasma membrane [17]–[19], we quantified the density of actin in the subplasmalemmal space under basal conditions by skeletonizing the actin network (see Materials and Methods) (Figure 5A; insets), resulting in 3.5±0.3 µm of total actin length/µm^2^ of cell image and 10.2±1.1 actin filament intersections/µm^2^ of cell image (Figure 5D). Following glucose stimulation for 2 to 10 minutes, during which time granule motion was more dynamic (see above), there was no apparent change in the actin appearance (Figure 5B) compared to basal conditions, as confirmed by unchanged total actin filament length (3.5±0.2 µm; p = 0.84) and number of actin filament intersections (10.8±1.5; p = 0.76) per µm^2^ of cell image (Figure 5B, 5D).

**Figure 6:**
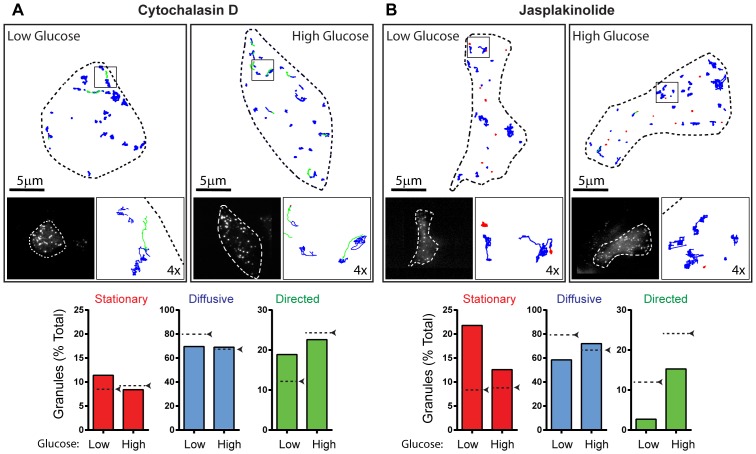
Effect of actin filament perturbation on insulin granule dynamics. (A and B: Upper Image Panels) Outline of a representative INS-1 cells with insulin granule trajectories overlaid in low and high glucose conditions after treatment with cytochalasin D (A) and jasplakinolide (B). Left inset: image of INS-1 cells with eGFP-labeled insulin granules. Right inset: 4× magnification of trajectories in the black box. Trajectories are color-coded to depict mode of granule motion. Red indicates stationary granule, blue indicates diffusive-like motion, and green indicates directed motion. (A and B: Lower Panel) Bar charts depict the percentage of granules exhibiting stationary, diffusive-like, and directed motions in low and high glucose conditions after cytochalasin D (A) and jasplakinolide treatment (B). Black arrows/dashed lines indicate the percentage of granules in each population in control cells. Cytochalasin D: 12 cells/230 granules in low glucose and 15 cells/289 granules in high glucose conditions were analyzed. Jasplakinolide: 7 cells/174 granules in low glucose and 6 cells/211 granules in high glucose conditions were analyzed.

To determine how pharmacologic perturbations to the actin cytoskeleton affected insulin granule trafficking, we first treated INS-1 cells with cytochalasin D as a means of depolymerizing actin filaments. As a result, little if any actin filaments remained in the cortex as observed visually and quantified by a 10-fold reduction in the total actin filament length and number of actin filament intersections per µm^2^ of cell image (Figures 5C, 5D). In the basal state, the greatest effect of cytochalasin D treatment on granule motion was a 50% increase in the proportion of directed runs, from 12–19%, although they moved with a slightly slower velocity (Figure 6A, Movie S7, Table 1). Upon glucose stimulation in these cytochalasin D treated cells, the percentage of granules undergoing directed motion increased only slightly to 22%; a value comparable to that observed in stimulated control cells, with similar velocities of 0.98±0.1 µm/s (Figure 6A; Table 1). Diffusion coefficients were 1.5-fold faster than in control cells increasing to 3093±115 nm^2^/s. Actin filament stabilization was accomplished by jasplakinolide treatment [45]. Although STORM images of the actin cytoskeleton were not possible due to jasplakinolide competing with phalloidin for actin binding [46], a dramatic 3-fold increase the percentage of stationary granules was observed under basal conditions and a decrease in the percentage of granules displaying directed motion to 3% (Figure 6B, Movie S8), the same proportion of directed runs observed after microtubule depolymerization with nocodazole (Figure 4B). Jasplakinolide-treated cells remained glucose responsive with the stationary granule population giving way to an increased percentage of granules that were directed (Figure 6B). However, the number of granules in the subplasmalemmal space did not decrease in response to glucose as in control cells (Figure 2B). Diffusion coefficients were significantly lower in jasplakinolide-treated cells than in control cells in both basal and glucose-stimulated conditions (Table 1).

## Discussion

The focus of our study was to determine how the spatial organization of both the actin and microtubule cytoskeletons contributes to insulin granule trafficking within 150 nm of the β-cell’s plasma membrane. This spatial region of the cell encompasses such critical processes as granule delivery by kinesin-1 motors on microtubules, distribution and retention of granules by myosin Va, and docking of granules to membrane fusion sites for eventual insulin secretion. Using live cell TIRF microscopy, we visualized the dynamic and complex movement of individual insulin granules within this subplasmalemmal space of INS-1 cells both before and after glucose stimulation. Insulin granule trafficking in live cells has been described previously, but usually a single global parameter, such as velocity or diffusion coefficient, was used to characterize overall granule motion [14], [22], [47]. However, as observed here, granules undergo 3 modes of movement (stationary, diffusive-like, directed), with the distribution between these modes sensitive to glucose stimulation (Figure 2A). To understand how the actin and microtubule cytoskeletons contribute to insulin granule dynamics, we used super-resolution STORM imaging to visualize these cytoskeletal networks at the single filament level (Figures 3, 5). Interestingly, subplasmalemmal dense actin filament and microtubule meshworks spatially coexist before and after glucose stimulation and by appearance alone, each cytoskeleton presents a dense meshwork of intersecting filaments that potentially creates either a physical barrier to granule delivery or a complicated highway system for molecular motor transport. By pharmacologically perturbing the actin and microtubule filamentous states, we conclude that the actin in the subplasmalemmal space restricts directed granule motion under basal conditions even though there appears to be no whole scale rearrangement or depolymerization of the actin cytoskeleton upon glucose stimulation. In addition, the significant increase in directed granule movements near the cell membrane upon stimulation is in fact microtubule-dependent and thus kinesin motors are most likely involved in the transport of granules within the subplasmalemmal space and not limited to long-range granule transport from the cell interior.

### The molecular basis of insulin granule movements

The molecular basis for granules appearing stationary or diffusive-like within the subplasmalemmal space are varied. For example, stationary granules may be simply docked to release sites on the plasma membrane or trapped within the actin cortex by the tethering capacity of both the myosin Va motors [48] and the putative myosin Va-granule adapter protein, exophilin 8 (MyRIP/Slac2-C), which itself can bind actin [49]. With regards to the diffusive-like granule population, several lines of evidence suggest that the random, diffusive-like movements are not solely thermal but may result from active, enzymatic processes as follows: (1) Qdot-labeled myosin Va motors introduced into COS-7 cells appear to undergo diffusion but in fact are randomly walking on the actin meshwork [37]; (2) granule diffusion coefficients decrease two fold with a 10 degree temperature change (Q_10_ = 2), which is indicative of an active process [12], and; (3) in permeabilized MIN-6 β-cells, diffusive-like granule motion is only observed in the presence of ATP [22]. Regardless of the underlying mechanism, the range of diffusion coefficients reported here (Table 1∶700–3,000 nm^2^/s) are within range of reported values for vesicles in various cell types [22], [50]–[53] but are 500–1000 times slower than predicted values for diffusion coefficients of insulin granule size beads in water [54]. The changes in diffusion coefficient observed after actin and microtubule stabilization and depolymerization collectively suggest that diffusive-like vesicle cargos must be experiencing a barrier created by both actin and microtubule cytoskeletons [51], [54], [55] as well as macromolecular crowding in the cytoplasm.

With regards to the directed granule motion, long range transport of insulin granules from the cell center to the periphery relies on kinesin-1 motion along microtubules [8], [56]. Given the presence of a dense microtubule network in close proximity to the cell membrane (Figure 3; Movies S2-4), this network most likely provides tracks for kinesin-1 directed granule movements within the subplasmalemmal space, since microtubule disruption with nocodazole (Figure 3D) almost completely eliminated directed granule movement (Figure 4B) [10]–[12]. In addition, velocities of directed granule transport were similar to single motor kinesin-1 velocities reported *in vitro* [13], [57], [58] and *in vivo* for kinesin-1 driven neuronal vesicles [59], [60].

### Cytoskeletal involvement in insulin granule movement

The cortical actin network may serve numerous roles in insulin granule motion dynamics. For example, the actin network may act as a track on which myosin Va molecular motors carry their granule cargo [6], [7], provide sites for vesicle tethering to restrict granule motion under basal conditions [49], or restrict granule docking to the plasma membrane by actin binding of t-SNAREs [18], [19]. Given that under basal conditions depolymerization of actin resulted in an increase in the proportion of granules undergoing directed motion and conversely, stabilization of the actin dramatically decreased directed granule motion and significantly increased the percentage of stationary granules, we suggest that our data are consistent with the actin network acting as a barrier to directed granule transport at least in low glucose, basal conditions.

Upon glucose stimulation, granule motion becomes more dynamic, i.e. diffusive-like granules have higher diffusion coefficients and a greater percentage of granules undergo directed motion (Figures 1A and 2A; Table 1). Our super-resolution STORM images of the β-cell’s actin and microtubule cytoskeleton in the subplasmalemmal space showed no apparent rearrangement between basal and glucose stimulated conditions. These data suggest that the increased percentage in directed granule runs once the cell is stimulated is likely due to a change in the activity of granule-associate proteins, such as kinesin-1, myosin Va or their adaptors. In support of this notion, stabilization of the actin and microtubule cytoskeletons by jasplakinolide and taxol, respectively, does not prevent granule mobilization in high glucose (Figure 4A and 6B). One potential explanation for the sudden increase in directed runs upon glucose stimulation is that kinesin motors are regulated and activated by dephosphorylation of the kinesin heavy chain in response to glucose [61]. In addition, the enhancement in directed runs near the plasma membrane would be aided by the observed increase in polymerization of microtubules following glucose stimulation (Figure 3F).

### How can insulin granules get delivered to the plasma membrane?

Contrary to previously proposed models which limited the role of microtubules to serve as tracks for kinesin-directed transport of granules from the cell center, our data suggest that microtubule-based granule transport may act in concert with any actin-based transport that occurs within the subplasmalemmal space. However, the actin network appears to restrict microtubule-based transport under basal conditions, when insulin secretion needs to be inhibited. Interestingly, recent reports suggest that microtubule organization and dynamics itself may spatially dictate plasma membrane sites of vesicle fusion [32], [62], [63]. So not only would microtubules provide tracks for granule delivery but may also be the last stop on the delivery pathway to the plasma membrane.

## Supporting Information

Movie S1 **Insulin granule dynamics in INS-1 cells expressing proinsulin-GFP under basal low conditions (left) and stimulatory, high glucose conditions (right).** Imaging speed 19 fps, play back 5x real time. Relates to main text figures 1 and 2.
(MOV)

Movie S2 **Z-stack (500** **nm/step) of microtubules labeled with mouse anti-tubulin and anti-mouse Alexa-660 in fixed INS-1 cell imaged using deconvolution epifluoresence microscopy.** Relates to main text figure 3.
(MOV)

Movie S3 **Microtubule dynamics in INS-1 cells expressing GFP-tubulin. Imaging speed 10** **fps, play back 6x real time.** Relates to main text figure 3.
(MOV)

Movie S4 **Dynamics of growing microtubules imaged in INS-1 cells expressing EB3-GFP under basal, low glucose conditions (left), stimulatory high glucose conditions (middle) and after taxol treatment (right).** Imaging speed 5fp, play back 10x real time. Relates to main text figure 3.
(MOV)

Movie S5 **Insulin granule dynamics in taxol-treated INS-1 cells expressing proinsulin-GFP under basal low conditions (left) and stimulatory, high glucose conditions (right). Imaging speed 19** **fps, play back 5x real time.** Relates to main text figure 4.
(MOV)

Movie S6 **Insulin granule dynamics in nocodazole-treated INS-1 cells expressing proinsulin-GFP under basal low conditions (left) and stimulatory, high glucose conditions (right).** Imaging speed 19 fps, play back 5x real time. Relates to main text figure 4.
(MOV)

Movie S7 **Insulin granule dynamics in cytochalasin D-treated INS-1 cells expressing proinsulin-GFP under basal low conditions (left) and stimulatory, high glucose conditions (right).** Imaging speed 19 fps, play back 5x real time. Relates to main text figure 6.
(MOV)

Movie S8 **Insulin granule dynamics in jasplakinolide-treated INS-1 cells expressing proinsulin-GFP under basal low conditions (left) and stimulatory, high glucose conditions (right).** Imaging speed 19 fps, play back 5x real time. Relates to main text figure 6.
(MOV)

File S1 (DOC)
