# Supplementary material for: Cytoskeletal Dependence of Insulin Granule Movement Dynamics in INS-1 Beta-Cells in Response to Glucose
Source: PLoS One. 2014 Oct 13;9(10):e109082. doi: 10.1371/journal.pone.0109082 (PMC4195697; doi:10.1371/journal.pone.0109082)
Supplement: File S1 — (DOC) [file pone.0109082.s009.doc]

# Supplemental Information

# Methods S1

**MSD Fitting.** To validate the tracking and MSD analysis approaches, as well as establish the basal behavior of truly immobile granules, we tracked granules in cells that were fixed in formaldehyde (see Materials and Methods). We found the MSD plots to often include an initial portion with a steeper slope, shown in **Fig S1A**), Since the MSD slope defines the diffusive exponent α, which is used to classify granule motion into stationary, diffusive-like and directed motions (see main text and supplemental section “determine alpha value cutoff values”), this initial steep portion of the MSD curve causes α values to range above 0.5, with a mean value of 0.24±0.11 (**Fig S1B**), even though the granules are immobile. However, by effectively down-sampling the data by omitting the shortest 1 or 2 time intervals from the fit (**Fig S1C & D**), we could more accurately characterize these immobile particles, with measured α values of 0.14±0.11 and 0.05±0.02, respectively. Therefore, to reduce the influence of the tracking noise on measured α values, all α values reported in this study are calculated omitting the initial 2 points from the MSD, effectively down sampling the data to 4.75Hz (from 19Hz).

# Determining Alpha cutoff values. To establish cutoff values to discriminate distinct modes of motion, we simulated 100-frame trajectories of each mode of motion that were parameterized to match the experimental frame rate (19Hz) and tracking precision (~30nm). These trajectories are each composed of a single mode of motion - stationary, diffusive-like (diffusion coefficient = 2600nm^2^/s) or directed runs (velocity = 1μm/s). Based on the resulting alpha distributions (shown in Fig S2), we identify directed runs having an α>1.5 (simulated α=1.9±0.01). However, under experimental conditions, diffusive-like motion appears as sub-diffusive (α = 0.7±0.2), even with the down-sampling of the data (as discussed above in section entitled “MSD Fitting”). This is largely attributable to the magnitude of the frame-to-frame motion (~24nm at 19Hz) being similar to the tracking error (~30nm). Accordingly, for this study we define diffusive-like motion to be 0.25<α<1.5, and stationary granules as α<0.25.

**ChangePoint Algorithm**. Many recorded trajectories contained visually apparent periods of distinct modes of motion (stationary, diffusive-like, or directed runs). Therefore, we sought an unbiased approach to segment these trajectories into periods of their component modes of motion. We adapted the method attributed to , which identifies a transition in the governing parameters as a minimum value of the Bayesian Information Criterion (BIC; ), a measure of goodness of fit, which accounts for both the sample size and the number of parameters used in the fit. For this approach, we consider changes in the diffusive exponent, often termed α , which describes the type of motion of a particle, which in this study is an individual insulin granule trajectory of paired x-y coordinates for *n* frames: (x,y)_1_, (x,y)_2,…_(x,y)_n_. (Example shown in **Fig. S3A**). To test for a transition occurring after frame *i*, we perform the following steps:

1) Calculate granule displacements over various time intervals as:

$d_{j,k}=(x_{k+j}-x_{k})^{2}+(y_{k+j}-y_{k})^{2}$ (Eq. 1)

for values of *k* from 1 to (i-j) (before the transition) and from (*i*+1) to *n* (after the transition) and values of the time interval, *j,* from 2 to (*k*-1). The selection of this range of the value *j* is discussed above in the section entitled “MSD Fitting.”

2) Lines are fit to log-log plots of squared displacement (*d*) vs time interval (*j*) for the intervals occurring before and after the transition (i.e., *k* < *i* and *k* > (*i*+1), example shown in **Fig S3D**).

3) The overall goodness-of-fit metric is then calculated as the sum of the BIC for both intervals (before and after the possible transition *i*).

(Eq. 2)

where *k* is the number of parameters for the fit (2), *n* is the number of measurements in the fit and σ_e_ is the error in the fit from Step 2.

Steps 1-3 are then repeated for all possible values of *i*, (example shown in **Fig S3B**). As this necessitates a measurable period of at least a few frames for each mode of motion, we only consider values of *i*, such that 5<*i*<(*n*-5). A transition is then determined to have occurred at the minimal value of the summed BIC, so long as this value lies below a cutoff of -0.4 (determined using modeled data, discussed below in the section “Determining BIC cutoff values”). Subsequent rounds test for additional transitions in the same manner, separately analyzing each segment of the now divided trajectory (example shown in **Fig S3C**). Finally, individual segments are classified as “Stationary,” “Diffusive-like,” or “Directed” based on their α values, utilizing the cutoffs discussed above in the section entitled “Determining Alpha Cutoff Values” and **Fig S2**.

**Determining BIC cutoff values.** To determine an appropriate cutoff BIC value to establish that a transition has occurred, we tested the algorithm using a panel of simulated trajectories that contain either 0 or 1 transitions in the mode of motion (e.g. from stationary to diffusive-like motion). For each trajectory, the minimum BIC value is determined as described above. Distributions of these minimum BIC values are shown in Fig S4 for trajectories that do (green) or do not contain (red) a transition in the mode of motion. Based on these two distributions, we determined that a value of -0.4 (dashed vertical line) provides the best separation between true transitions and potential false positives. However, this cutoff still misses 5% of true transitions and inappropriately detects transitions in 4% of trajectories where no transition is present.

**Supplemental Figures**

**
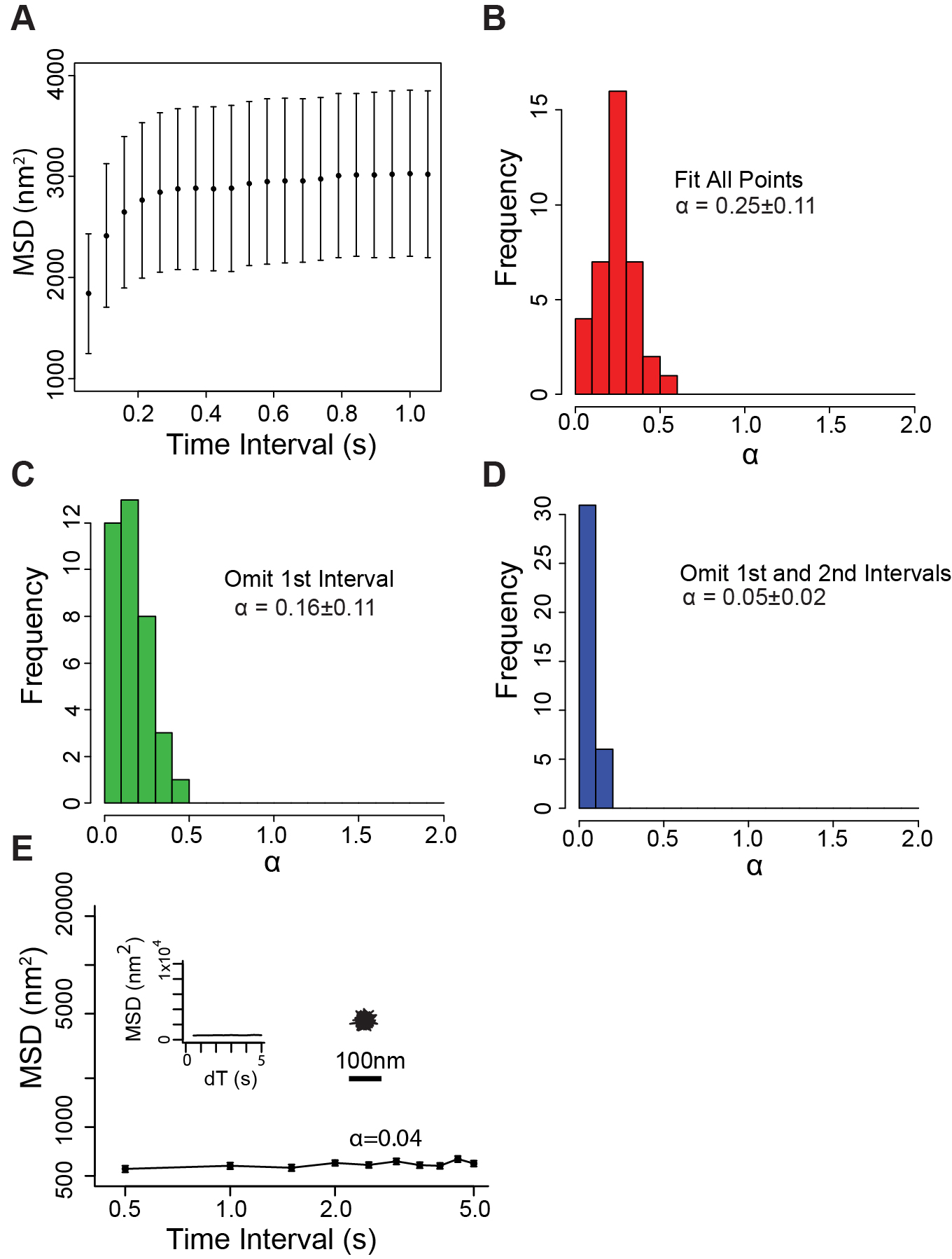
**

**Figure S1.** (A) Sample MSD plot of insulin granules in fixed cells on a linear scale. (B) Frequency histogram of alpha values when all points of MSD plot are used to calculate alpha. (C and D) Frequency histograms of alpha values when the shortest 1 (C) or 2 (D) time intervals are omitted from MSD calculation. (E) Example trajectory of insulin granule in fixed cells and MSD plots on log-log axes (large graphs) and linear axes (inset) are shown when first two time intervals are omitted from calculation. Relates to main text figure 1.

**
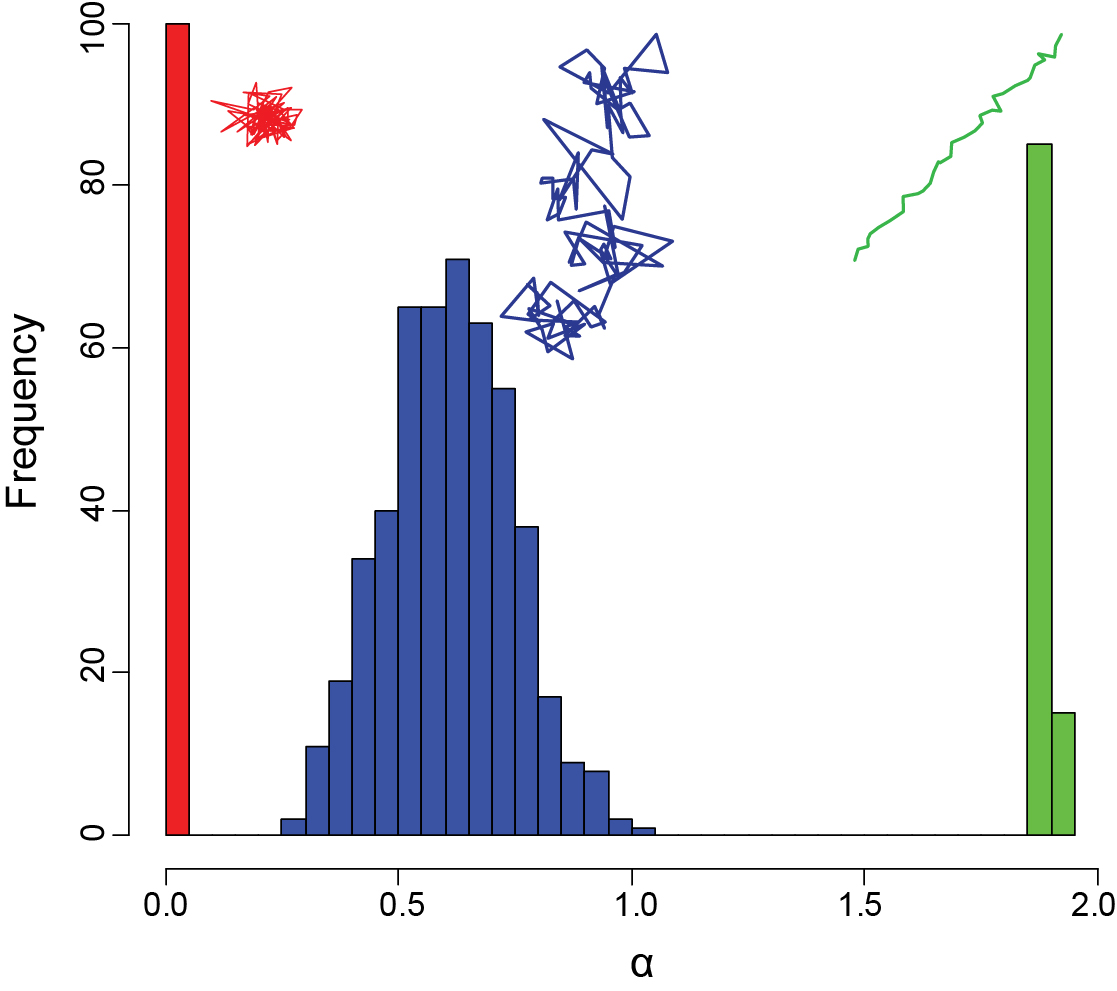
**

**Figure S2.** Alpha values of simulated trajectories. Example of simulated trajectories and frequency distribution of alpha values for stationary (red), diffusive-like (blue) and directed (green) granule motion. Relates to main text figure 1.

**
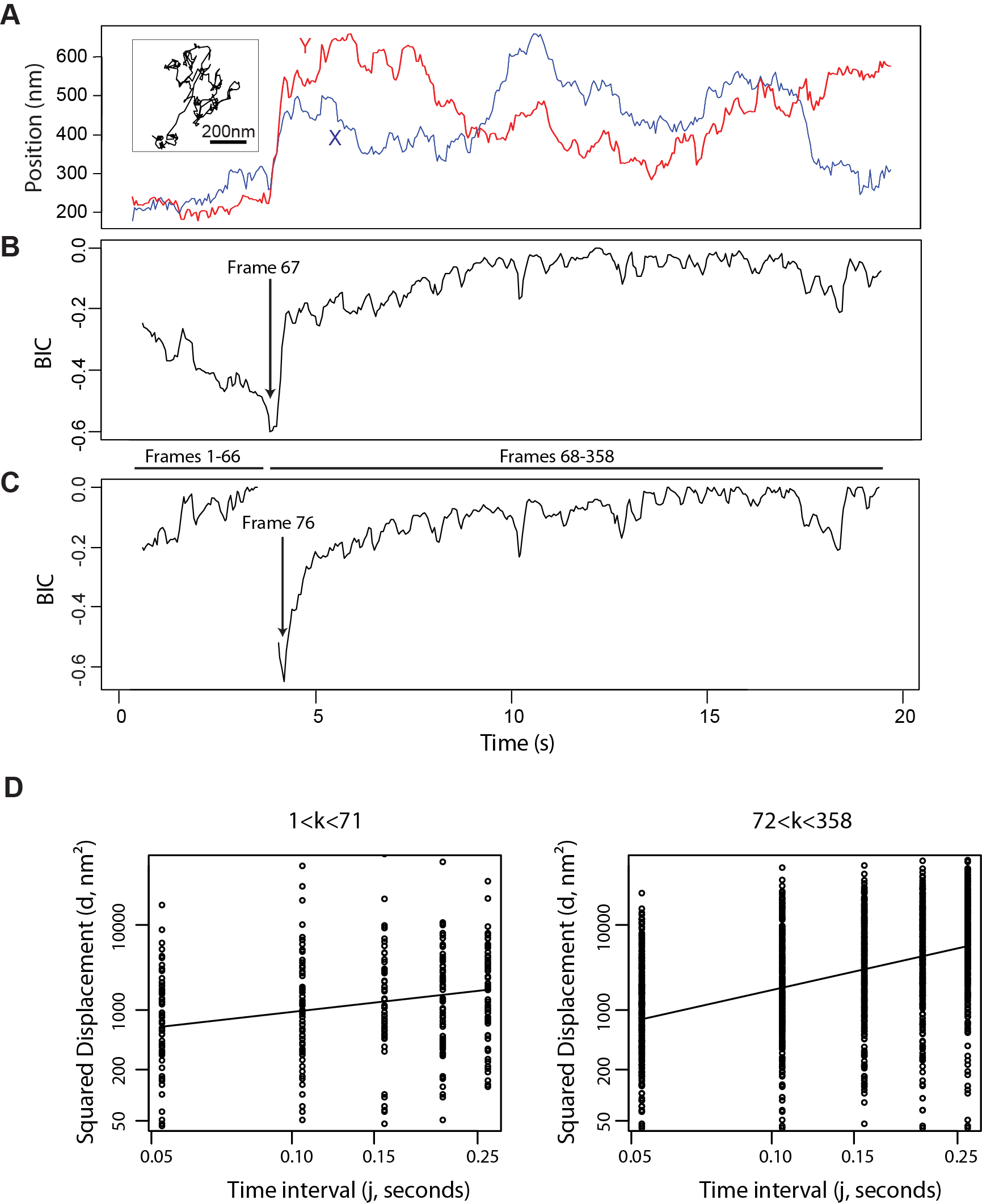
**

**Figure S3**. Workflow for ChangePoint algorithm. (A) trajectory of a single insulin granule under stimulatory, high glucose conditions is shown as time courses for x- (blue) and y-axis (red) position (large graph), and combined 2-D trace (inset). (B) Scanning over the range of possible transitions (1<*k*<358 for this trajectory) an optimal fit (minimal value) is shown at frame 67. (C) Subsequent rounds check for additional ChangePoints over the intervals 1<*k*< 67 and 68<*k*<358, detecting an additional transition at frame 76. A third scan does not detect any values below the pre-determined cutoff value of -0.4 (see **Fig. S4** and supplemental methods section “Determining BIC cutoffs”), therefore, this approach identifies 2 transitions for this trajectory at frames 67 and 76. The resulting segments have alpha values of 0.52, 1.93, and 1.06 (main text **Fig. 1D**). (D) For a candidate transition point (*i*), after frame 71 in this example, the goodness of fit measure is calculated as the summed BIC for the two linear regressions to squared displacement data versus time interval (*j*) both before (left) and after (right) of the candidate transition. Relates to main text figure 1.


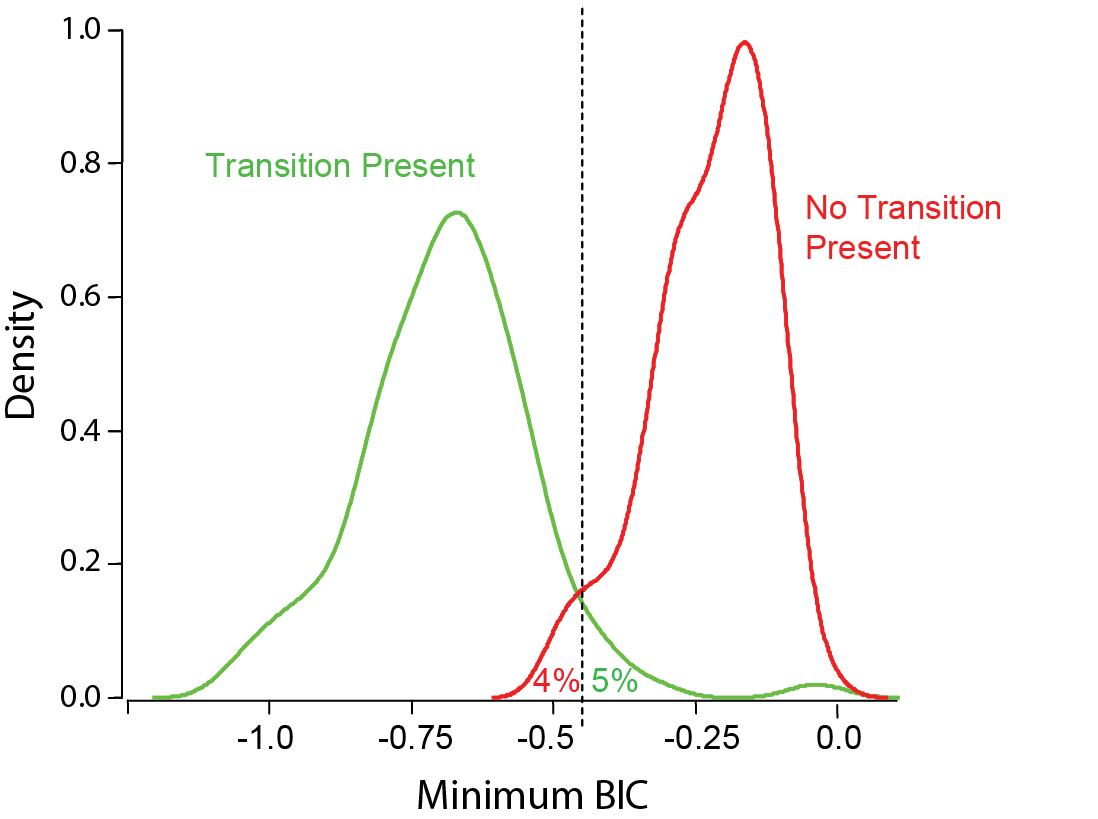


**Figure S4.** Frequency distribution of minimum BIC values for simulated trajectories with 0 (red) or 1 (green) transition. A minimum BIC value of -0.4 (dashed line) was determined to be best separation between the trajectories with a true transition and potential false positives. This cutoff misses 5% of true transitions and inappropriately detects transitions in 4% of trajectories that do not contain a transition. Relates to main text figure 1.

**Supplemental Movies**

Movie S1: Insulin granule dynamics in INS-1 cells expressing proinsulin-GFP under basal low conditions (left) and stimulatory, high glucose conditions (right). Imaging speed 19fps, play back 5x real time. Relates to main text figures 1 and 2.

Movie S2: Z-stack (500nm/step) of microtubules labeled with mouse anti-tubulin and anti-mouse Alexa-660 in fixed INS-1 cell imaged using deconvolution epifluoresence microscopy. Relates to main text figure 3.

Movie S3: Microtubule dynamics in INS-1 cells expressing GFP-tubulin. Imaging speed 10fps, play back 6x real time. Relates to main text figure 3.

Movie S4: Dynamics of growing microtubules imaged in INS-1 cells expressing EB3-GFP under basal, low glucose conditions (left), stimulatory high glucose conditions (middle) and after taxol treatment (right). Imaging speed 5fp, play back 10x real time. Relates to main text figure 3.

Movie S5: Insulin granule dynamics in taxol-treated INS-1 cells expressing proinsulin-GFP under basal low conditions (left) and stimulatory, high glucose conditions (right). Imaging speed 19fps, play back 5x real time. Relates to main text figure 4.

Movie S6: Insulin granule dynamics in nocodazole-treated INS-1 cells expressing proinsulin-GFP under basal low conditions (left) and stimulatory, high glucose conditions (right). Imaging speed 19fps, play back 5x real time. Relates to main text figure 4.

Movie S7: Insulin granule dynamics in cytochalasin D-treated INS-1 cells expressing proinsulin-GFP under basal low conditions (left) and stimulatory, high glucose conditions (right). Imaging speed 19fps, play back 5x real time. Relates to main text figure 6.

Movie S8: Insulin granule dynamics in jasplakinolide-treated INS-1 cells expressing proinsulin-GFP under basal low conditions (left) and stimulatory, high glucose conditions (right). Imaging speed 19fps, play back 5x real time. Relates to main text figure 6.

# Supplemental References

1. Hamilton, *A new approach to the economic analysis of nonstationary time series and the business cycle.* Econometrica, 1989. **57**: p. 357-384.

2. Schwartz, G., *Estimating the dimension of a model.* Annals of Statistics 1978. **6**(2): p. 461-464.

3. Saxton, M.J. and K. Jacobson, *Single-particle tracking: applications to membrane dynamics.* Annu Rev Biophys Biomol Struct, 1997. **26**: p. 373-99.
